# Genetic Analysis of *ABCB4* Mutations and Variants Related to the Pathogenesis and Pathophysiology of Low Phospholipid-Associated Cholelithiasis

**DOI:** 10.3390/genes13061047

**Published:** 2022-06-11

**Authors:** Helen H. Wang, Piero Portincasa, Min Liu, David Q.-H. Wang

**Affiliations:** 1 Department of Medicine and Genetics, Division of Gastroenterology and Liver Diseases, Marion Bessin Liver Research Center, Einstein-Mount Sinai Diabetes Research Center, Albert Einstein College of Medicine, Bronx, NY 10461, USA; helen.jiang@einsteinmed.edu; 2 Department of Biomedical Sciences and Human Oncology, Clinica Medica “A. Murri”, University of Bari Medical School, 70124 Bari, Italy; piero.portincasa@uniba.it; 3 Department of Pathology and Laboratory Medicine, University of Cincinnati College of Medicine, Cincinnati, OH 45237, USA; lium@ucmail.uc.edu

**Keywords:** ABC transporters, bile acids, bile flow, bile formation, biliary lipid secretion, cholesterol crystallization, gallstones, gene mutations and variants, hepatocytes, *Lith* genes, micelle, vesicle

## Abstract

Clinical studies have revealed that the *ABCB4* gene encodes the phospholipid transporter on the canalicular membrane of hepatocytes, and its mutations and variants are the genetic basis of low phospholipid-associated cholelithiasis (LPAC), a rare type of gallstone disease caused by a single-gene mutation or variation. The main features of LPAC include a reduction or deficiency of phospholipids in bile, symptomatic cholelithiasis at <40 years of age, intrahepatic sludge and microlithiasis, mild chronic cholestasis, a high cholesterol/phospholipid ratio in bile, and recurrence of biliary symptoms after cholecystectomy. Needle-like cholesterol crystals, putatively “anhydrous” cholesterol crystallization at low phospholipid concentrations in model and native bile, are characterized in ABCB4 knockout mice, a unique animal model for LPAC. Gallbladder bile with only trace amounts of phospholipids in these mice is supersaturated with cholesterol, with lipid composition plotting in the left two-phase zone of the ternary phase diagram, consistent with “anhydrous” cholesterol crystallization. In this review, we summarize the molecular biology and physiological functions of ABCB4 and comprehensively discuss the latest advances in the genetic analysis of *ABCB4* mutations and variations and their roles in the pathogenesis and pathophysiology of LPAC in humans, based on the results from clinical studies and mouse experiments. To date, approximately 158 distinct LPAC-causing *ABCB4* mutations and variants in humans have been reported in the literature, indicating that it is a monogenic risk factor for LPAC. The elucidation of the ABCB4 function in the liver, the identification of *ABCB4* mutations and variants in LPAC patients, and the exploration of gene therapy for ABCB4 deficiency in animal models can help us to better understand the cellular, molecular, and genetic mechanisms underlying the onset of the disease, and will pave the way for early diagnosis and prevention of susceptible subjects and effective intervention for LPAC in patients.

## 1. Introduction

Bile is an aqueous solution and contains three major lipids such as cholesterol, phospholipids, and bile acids, with bile pigments being a minor solute [1]. Cholesterol is a major sterol in bile, accounting for roughly 95% of total sterols. Lecithin, also called phosphatidylcholine, comprising more than 95% of total phospholipids, is an insoluble, swelling amphiphile with a hydrophilic, zwitterionic phosphocholine head group and two hydrophobic long fatty acyl chains [2,3,4,5]. By weight, bile acids make up about two thirds of the solute mass in normal human bile and are a family of closely related acidic sterols that are synthesized from cholesterol in the liver through two pathways, i.e., the classical or neutral and the alternative or acidic pathways [6,7,8]. In humans, cholic acid with three hydroxyl groups at the C-3, C-7, and C-12 positions and chenodeoxycholic acid with two hydroxyl groups at the C-3 and C-7 positions are the two primary bile acids synthesized exclusively in the liver [9,10,11].

Bile formation is an extremely complex process involving multiple transporters for hepatic secretion of biliary lipids [12]. Because of the possession of both hydrophilic and hydrophobic surfaces, bile acids display a detergent-like amphiphilic property and are highly soluble in bile [13]. Due to their high water solubility, bile acids show the ability to self-assemble into micelles when they reach above the critical micellar concentration [14]. As bile is gradually concentrated in the biliary tree from the bile canaliculi to intrahepatic bile ducts, then to extrahepatic bile ducts, and eventually to the gallbladder, the concentration of bile acids steadily exceeds its critical micellar concentration, allowing them to form simple micelles in bile [15,16,17,18]. Notably, simple micelles can also dissolve other types of lipids, such as cholesterol and phospholipids, by forming mixed micelles in bile. Although lecithin is insoluble in water, it can form unilamellar vesicles with membrane bilayers containing mainly lecithin and cholesterol with only trace amounts of bile acids, if any [19,20,21]. Therefore, compared to micelles, vesicles can dissolve even more cholesterol molecules in bile. Unilamellar vesicles can fuse to form large multilamellar vesicles, also known as liposomes or liquid crystals [22]. Because cholesterol is actually insoluble in water, the mechanisms by which cholesterol dissolves in bile are complex. Therefore, cholesterol must be transported in bile along with bile acids and phospholipids, and both micelles and vesicles are the two major macromolecular aggregates in bile, also known as cholesterol carriers [23]. In diluted bile with total lipid concentrations <3 g/dL, vesicles are stable and neither aggregate, fuse nor nucleate cholesterol. However, in concentrated gallbladder bile at approximately 10 g/dL, vesicular instability is significantly increased, and the precipitation of solid cholesterol crystals from vesicles is greatly accelerated [24]. It has been proposed that solid cholesterol crystals can nucleate from these multilamellar vesicles in concentrated gallbladder bile [25,26,27,28]. However, it remains elusive whether cholesterol can nucleate from simple or mixed micelles in bile.

**Figure 1:**
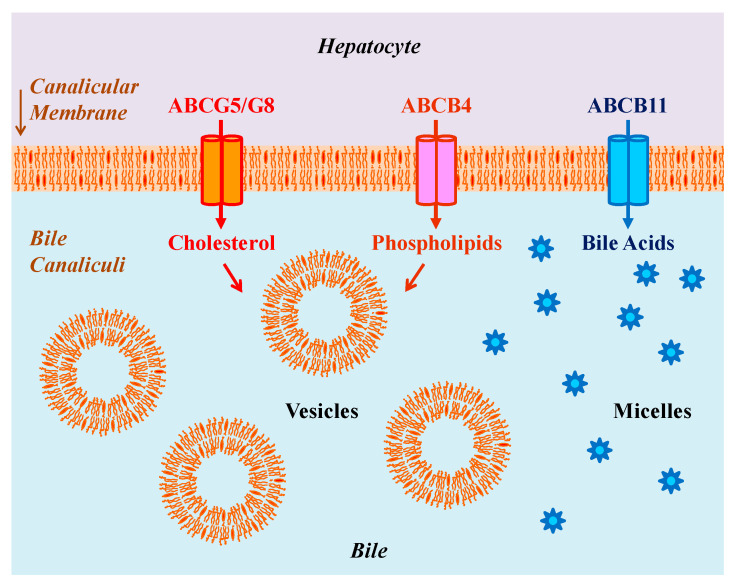
During bile formation, cholesterol, phospholipids, and bile acids are secreted by ABCG5/G8, ABCB4, and ABCB11 transporters, respectively, from the hepatocytes into the bile canaliculi. These three lipid transporters are located on the canalicular membrane of hepatocytes. After being secreted into bile, phospholipids often form unilamellar vesicles (~40 to 100 nm in diameter) that are a spherical structure, i.e., a single bilayer that encircles an aqueous core. Because bile is an aqueous solution and cholesterol is virtually insoluble in water, biliary vesicles play a major role in the transport of cholesterol in bile. In addition, bile acids, which are synthesized in the liver, can self-assemble to form simple and mixed micelles in bile. Notable, these micelles can also solubilize cholesterol in bile (not shown). Abbreviation: ABC, adenosine triphosphate (ATP)-binding cassette (transporter).

Hepatic secretion of three major biliary lipids across the canalicular membrane of hepatocytes is determined exclusively by a group of members of the adenosine triphosphate (ATP)-binding cassette (ABC) transporter family (Figure 1). The sterol efflux transporters, ABCG5/G8, the bile acid export pump, ABCB11, and the phospholipid flip-flop transporter, ABCB4, play an essential role in the regulation of biliary cholesterol, bile acid, and phospholipid secretion, respectively [12]. Genetic analysis has clearly demonstrated that due to gene mutations and variations, dysfunction of one of these transporters can cause hepatobiliary and cardiovascular diseases [29,30,31]. For example, inherited deficiency of *ABCB11* due to gene mutations can result in progressive familial intrahepatic cholestasis type 2 (PFIC2) [32,33,34]. Epidemiological investigations and clinical studies have found that PFIC2 is a chronic liver disease and can progress to liver fibrosis and eventually to cirrhosis, and it often necessitates liver transplantation in early childhood [35]. Furthermore, mutations in either *ABCG5* or *ABCG8*, but not simultaneously, can cause a rare autosomal recessively inherited lipid metabolic disorder called sitosterolemia [36,37,38]. Patients with sitosterolemia show a dramatic reduction in biliary cholesterol secretion and a significant increase in intestinal absorption of cholesterol and plant sterols, also called phytosterols, as well as suffer from hypercholesterolemia, premature development of atherosclerosis, tendon and tuberous xanthomas, and abnormal hematologic and liver functions [39,40,41]. On the contrary, as found by a genome-wide association study in a large cohort of patients with gallstones, as well as by a linkage study in affected sibling pairs, several variants in either *ABCG5* or *ABCG8* are associated with hepatic cholesterol hypersecretion, thereby leading to the development of cholesterol-supersaturated gallbladder bile and the formation of gallstones [42,43]. In addition, genetic factors of cholesterol gallstone disease have been extensively investigated in inbred strains of mice by quantitative trait locus (QTL) methods [44,45,46], and gallstone and biliary phenotypes have been comprehensively studied in these inbred mice. The *Abcg5/g8* has been identified as *Lith9* on mouse chromosome 17 by the QTL studies [47,48,49]. Subsequently, some clinical studies have revealed that *ABCG5/G8* is also a human gallstone gene, *LITH9*, in European, Asian, and Chilean Hispanic populations [43,50,51,52,53].

Notably, ATP-binding cassette, sub-family B (MDR/TAP), member 4 (ABCB4), also known as multidrug resistance protein 3 (MDR3 in humans and Mdr2 in mice), is the membrane-associated transport protein almost exclusively expressed in the liver [54]. *ABCB4* encodes the hepatic phosphatidylcholine floppase that plays a key role in determining biliary phospholipid secretion because it can translocate the major species of biliary phospholipids, i.e., lecithin or phosphatidylcholine, from the inner to the outer leaflet of the canalicular membrane of hepatocytes for secretion into bile [55,56,57]. This floppase activity enables the secretion of phospholipids into the bile canaliculi. Therefore, dysfunction of ABCB4 can dramatically reduce hepatic phospholipid secretion, thereby leading to toxic membrane damage by an excess of biliary bile acids due to their detergent and cytotoxic properties [58]. It has been recognized that ABCB4 deficiency caused by genetic mutations or variations mainly involves three major hepatobiliary diseases: progressive familial intrahepatic cholestasis type 3 (PFIC3), intrahepatic cholestasis of pregnancy (ICP), and low phospholipid-associated cholelithiasis (LPAC) [59]. In these hepatobiliary disorders associated with ABCB4 deficiency, the lipid composition of bile is primarily characterized by a dramatic reduction in phospholipid concentrations or a lack of biliary phospholipids, often accompanied by relatively normal bile acid concentrations.

In the present review, we briefly summarize the currently available knowledge about the molecular biology and physiological functions of ABCB4, as well as systematically highlight the latest advances in the genetic analysis of *ABCB4* mutations and variations and their roles in the pathogenesis and pathophysiology of LPAC in humans, primarily based on the results from clinical studies and mouse experiments. Until now, nearly 158 distinct LPAC-causing *ABCB4* mutations and variants in humans have been identified. Understanding fundamental ABCB4 function in the liver, identifying new *ABCB4* mutations and variants in patients with LPAC, and investigating potential gene therapy for ABCB4 deficiency in animal models should provide novel insights into the cellular, molecular, and genetic mechanisms underlying the onset and course of the disease, and will eventually open the door for early diagnosis and prevention of susceptible subjects and effective intervention for LPAC in patients.

## 2. Identification of *ABCB4* in Mice and Humans

The physiological function of mammalian P-glycoproteins was unknown when it was first identified [60]. The human *MDR1* gene is expressed in many tissues of the body, most prominently in the epithelia of excretory organs. This fits the hypothesis that the MDR1 P-glycoprotein may play a role in the elimination of toxic metabolites or xenobiotic compounds from the body [61,62,63]. However, expression of the human *MDR3* gene is much more restricted. Substantial amounts of human *MDR3* mRNA have thus far only been found in the liver and in prolymphocytic leukemia cells of the B-cell lineage [64]. The name *MDR3* was originally assigned because of the high homology because of 78% identical residues with the ABC transporter P-glycoprotein coded by the multidrug resistance 1 (*MDR1*) gene, also called *ABCB1* gene, which shares the same domain organization with *MDR3* [56]. These transporters contain four distinct protein domains, and their ancestor appears to have arisen by gene duplication [62].

When first discovered, ABCB4 was assumed to be a multidrug exporter due to its high sequence conservation with 76% identity and 86% similarity to ABCB1, a P-glycoprotein, and ABCB4 is still often referred to as multidrug resistance protein 3 (MDR3) for humans [65,66,67]. However, various studies have shown that ABCB4 was capable of recognizing and transporting some ABCB1 substrates, albeit with low capacity, and the data regarding its role in conferring drug resistance were inconclusive [55,68]. Moreover, ABCB1 has only been shown to flip short-chain, fluorescently labeled lipid analogs in cellular assays and, despite its canalicular expression, ABCB1 is unable to compensate for the loss of ABCB4 function [69]. Consequently, it is now accepted that, despite their high sequence identity, ABCB4 and ABCB1 are functionally distinct proteins [57]. However, although the studies on multiple structures of ABCB1 have increased our mechanistic understanding of multidrug export [70], the results are insufficient to decipher the molecular details of ABCB4-catalyzed phospholipid transport. Direct visualization of ABCB4 has been challenging, partly due to difficulties in obtaining suitable amounts of functional and pure ABCB4, possibly stemming from toxicity induced by its overexpression in mammalian cells. This has also hampered the efforts at purifying the protein, which could explain the limited number of in vitro studies on purified ABCB4.

**Figure 2:**
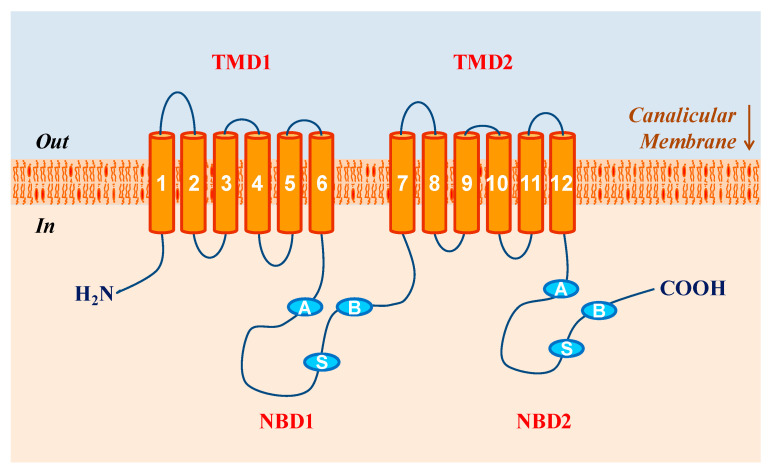
Schematic diagram of the adenosine triphosphate (ATP)-binding cassette (ABC) transporter ABCB4 that is encoded by the *ABCB4* gene and is located on Chromosome 7 (q21.1) in humans and on Chromosome 5 (3.43 cM) in mice, consisting of 27 coding exons and spanning approximately 74 kilobases (kb) in length. The orange cylinders symbolize the transmembrane domains (TMD) of the ABCB4 protein. TMD1 and TMD2 represent the two symmetrical regions that contain the transmembrane domains 1–6 and 7–12, respectively. Two TMDs are connected by six intracellular segments 1–6 and by six extracellular loops 1–6 (not shown). The *ABCB4* gene promoter harbors a CpG island that is usually unmethylated in the hepatocytes. The Walker A (A) and Walker (B) motifs and the signature (S) in the nucleotide binding domains (NBD) are indicated. NBD1 and NBD2 denote the nucleotide binding domains 1 and 2, respectively.

PFIC3 is caused by mutations of the *ABCB4* gene, a member of the superfamily of ABC transporters [71]. This ubiquitous molecular family uses energy derived from ATP hydrolysis to efflux a wide range of substrates across the cell membrane. ABCB4 polypeptide chain is organized into two repeats, each containing approximately 610 amino acid long and joined by a 60-residue linker region [72]. Moreover, each repeat has two structural modules, a transmembrane domain composed of six transmembrane α-helices and a cytoplasmatic nucleotide-binding domain [73]. On the cytoplasmatic side of the protein, four small linker peptides, also referred to as intracellular domains, hook up the transmembrane helices, and on the extracellular side, six short loops attach transmembrane segments (Figure 2). The two transmembrane domains contain specific sites for substrate binding and translocation, whereas the two nucleotide-binding domains, which display a high degree of sequence similarity with the equivalent domain of ABC transporters, couple the energy obtained from ATP hydrolysis for substrate transport [74]. The intracellular domains are deemed to be involved in mediating the coupling between the conformational changes of nucleotide-binding domain and the reorientation of transmembrane helices concomitant with substrate extrusion [75].

The human *ABCB4* gene is located on Chromosome 7 (q21.12) and is nearly 74 kilobases (kb) in length (GenBank accession number CH236949, region 10391396y10464818). It contains 27 coding exons, and the B isoform of the transcript encodes a polypeptide of 1,286 amino acids in length [72]. Furthermore, the *Abcb4* gene is found to be located on Chromosome 5 (3.43 cM) in mice, Chromosome 4 (q12) in rats, and Chromosome 16 in zebrafish.

In the early 1990s, Smit and colleagues [76] first found that hepatic phospholipid secretion is a protein-mediated process because deletion of the *Abcb4* gene in mice completely inhibits hepatic phospholipid secretion. As a result, it dramatically reduces the formation and secretion of vesicles on the outer surface of the canalicular membrane of hepatocytes [77]. This indicates that ABCB4 plays a critical role in the translocation, or “flip” of phosphatidylcholine from the endoplasmic (inner) to ectoplasmic (outer) leaflet of the canalicular membrane bilayer of hepatocytes, leading to the formation of phosphatidylcholine-rich microdomains within the outer membrane leaflet for hepatic phospholipid secretion [78]. Notably, the ectoplasmic leaflet of the canalicular membrane of hepatocytes is usually enriched with cholesterol and sphingomyelin, which is quite resistant to penetration by bile acids [79]. Thus, bile acids may interact with the canalicular membrane of hepatocytes and partition preferentially into these areas, promoting hepatic secretion of phosphatidylcholine-rich vesicles by destabilizing the membrane because of detergent-like properties of bile acids [80]. PFIC3 is patients is caused by a variety of mutations in the *ABCB4* gene [81]. In addition, *ABCB4* mutations and variants also result in LPAC, a rare biliary disease induced by a single-gene mutation or variation.

## 3. Molecular Biology and Physiological Function of ABCB4

The structure of the *ABCB4* gene is similar between humans and mice. The *ABCB4* gene spans roughly 74 kb in humans and about 68 kb in mice. Moreover, the *ABCB4* gene consists of 28 exons in humans and mice, with 27 exons containing coding sequences that correlate with functional domains. Introns are positioned at homologous sites within the coding regions, but only loosely match when N- and C-terminal halves are compared. Notably, ABCB4 consists of six intracellular domains and six extracellular loops separated by twelve transmembrane domains (Figure 2). The ABCB4 protein contains two intracytoplasmic ATP-binding domains, also known as nucleotide binding domains, with characteristic motifs Walker A, Walker B, and ABC signature located upstream from the Walker B domain. The nucleotide binding domains may transfer energy to transmembrane transport of the substrate against a concentration gradient, whereas the transmembrane domains provide specificity for the substrate such as phosphatidylcholine of the phospholipid family. Notably, expression of ABCB4 in the fetal liver is approximately 16-fold lower than that in the normal adult liver, suggesting that ABCB4 may develop late in gestation or even postnatally.

As evidenced by immunohistochemical staining of human and animal liver tissues, the ABCB4 protein is mainly expressed on the canalicular membrane of hepatocytes as it may be trafficked to the canalicular membrane that is functionally used as the boundary of the bile canaliculi. More importantly, the ABCB4 transporter is a phospholipid translocator essential for hepatic secretion of phospholipids into the bile canaliculi, favoring the formation of bile [82,83,84]. Moreover, its physiological function is closely coupled with the functions of two other ABC transporters, the bile salt export pump, ABCB11, and the heterodimeric sterol transporter, ABCG5/G8, for hepatic secretion of biliary bile acids and cholesterol, respectively [85]. The phospholipid secretion of ABCB4 is triggered by the canalicular bile acids secreted by ABCB11, together with the cholesterol secretion mediated by ABCG5/G8 [86,87,88]. Balancing the hepatic transport processes of these three major biliary lipids is critical to maintain these biliary constituents in an appropriate ratio for the assembly of simple and mixed micelles, as well as unilamellar and multilamellar vesicles, which is extremely important to protect the hepatocytes and the epithelial cells of bile ducts from the detergent-like cytotoxic property of certain hydrophobic bile acids [71].

Similar to other members of the ABC superfamily, ABCB4 can use energy obtained from ATP hydrolysis to transport specific substrates such as phospholipids across the canalicular membrane of hepatocytes into bile [89]. The results from mouse experiments have suggested that the phospholipid molecules may be translocated by ABCB4 from the inner to the outer leaflet of the canalicular membrane of the hepatocytes for hepatic secretion. As a result, the ABCB4-mediated translocation of phospholipids in the liver is known as the floppase activity, which makes the phospholipid molecules available for sustained extraction into the canalicular lumen by bile acids for the formation of bile [69]. Therefore, ABCB4 acts as an energy-dependent “floppase”, translocating phospholipids from the inner to the outer leaflet of the lipid bilayer of the canalicular membrane for hepatic secretion. The floppase activity of ABCB4 in the liver has been found in mice because deletion of the *Abcb4* gene disrupts hepatic phospholipid secretion mostly by impairing floppase activity. In addition, ABCB4 mediates the translocation of phospholipids across the canalicular membrane of the hepatocytes, a crucial process in protecting the cell membranes of hepatocytes and cholangiocytes from being exposed to high concentrations of detergent bile acids [90]. Absence or dysfunction of ABCB4 protein also causes the production of toxic bile characterized mainly by low phospholipid content, and sometimes, by high cholesterol levels [91]. Furthermore, the lack of phospholipids in bile markedly undermines protection against the detergent-like effect of bile acids, thus resulting in injury and damage to the epithelial cells of bile ducts, and subsequently promoting bile duct proliferation, progressive portal fibrosis, and eventually cirrhosis [92]. In addition to the lack of phospholipids in bile, all these abnormalities can cause liver fibrosis and cirrhosis in ABCB4 KO mice, with its liver pathology being similar to that of PFIC3 patients [93].

In hepatic bile, bile acids, phospholipids, and cholesterol often form mixed micelles [15]. Disrupted assembly of mixed micelles and subsequent accumulation of nonmicellar bile acids result in the production of bile with deleterious detergent property on the cell membrane of hepatocytes and cholangiocytes [94]. More importantly, biliary phospholipids play a key role in maintaining the solubility of cholesterol in bile [95], thus preventing its nucleation, crystallization, and precipitation, as well as subsequent formation of solid cholesterol crystals and gallstones, which will be discussed in detail in the next section.

The expression of *ABCB4* is regulated, at several levels, by transcriptional and posttranscriptional mechanisms [96]. The nuclear receptor, farnesoid X receptor (FXR), plays a decisive role in regulating and coordinating bile acid, cholesterol, triglyceride, glucose, and energy metabolism in the body. FXR also plays an essential role in the regulation of *ABCB4* expression and function [97]. Moreover, *ABCB4* may be trans-activated by FXR through a direct binding of FXR/retinoid X receptor-α (RXRα) heterodimer to a highly conserved inverted repeat-1 motif at the distal promoter [98]. Apart from bile acid-mediated activation of *ABCB4* through the FXR signaling cascade, fibric acid derivatives, such as fibrates, may stimulate *ABCB4* expression through the pathway of peroxisome proliferator-activated receptor α (PPARα), thus promoting hepatic phospholipid secretion [99].

In addition, the phospholipid molecule has been recognized as an endogenous ligand of the orphan nuclear receptor such as the liver receptor homologue-1 (LRH1), also called the *NR5A2* [100]. The results from animal studies have shown that LRH1 plays a critical role in the transcriptional regulation of *ABCB4* expression and accordingly affects hepatic secretion of biliary phospholipids [101]. Additionally, the ABCB4 transporter may act as a modulator of glucose metabolism and such an effect might also be mediated likely via the LRH1-dependent phospholipid pathway [102].

Despite its importance in liver health and disease, the high-resolution structure of ABCB4 remains unavailable for structural biology analysis. Due to the significant mechanistic diversity within the ABC transporter family, it is not possible to make mechanistic inferences from structural data of other ABC transporters. It has been proposed that ABCB4 may work by “flopping” the phospholipid molecules from the inner to the outer membrane leaflet of the canalicular membrane of hepatocytes, where the resulting excess of outer leaflet phospholipids is released into the canalicular bile in a process facilitated by the canalicular bile acids. However, it remains unclear how ABCB4 achieves the translocation and reorientation of phospholipids or whether the release of phospholipids is into the outer membrane leaflet or directly into the canalicular bile. Furthermore, other ABC transporters of lipid substrates traffic these in a variety of ways, with distinct mechanisms proposed for ABCA1, PglK, and MsbA.

Several *ABCB4* mutations have been examined in cellular assays to discern whether they may influence the function of the transporter by modulating protein expression or trafficking [103]. In addition to genetic mutation, ABCB4 expression and function can be inhibited by pharmaceutical compounds, leading to drug-induced liver injury [104]. Several marketed drugs with the risk profiles for drug-induced liver injury inhibit ABCB4 in cellular assays. For example, ABCB4 inhibition by Itraconazole has been shown to cause cholestasis in rats. In addition, genomic studies suggest a link between ABCB4 dysfunction and possible hepatobiliary malignancies, and it has been reported that *ABCB4* is frequently epigenetically silenced in hepatocellular carcinoma [93].

## 4. Role of ABCB4 in the Pathogenesis of Low Phospholipid-Associated Cholelithiasis

As mentioned above, biliary phospholipids play an important role in solubilizing excess cholesterol in the form of unilamellar and multilamellar vesicles (Figure 1). In agreement with this paradigm, ABCB4 deficiency can significantly inhibit hepatic secretion of biliary phospholipids, thereby leading to a dramatic decrease in phospholipid concentrations in bile [82,83,84]. As a result, the solubility of cholesterol in bile is markedly reduced, allowing for rapid cholesterol nucleation and crystallization for the formation of solid needle-like cholesterol crystals in the bile ducts and gallbladder of ABCB4 KO mice [105], as well as in model bile with low phospholipid concentrations [24,106,107].

In 2001, Rosmorduc and colleagues [108] first reported a clinical entity that is primarily characterized by the occurrence of symptomatic cholesterol gallstones in the gallbladder and intrahepatic bile ducts in young adults associated with *ABCB4* mutations. This rare form of cholesterol cholelithiasis, called LPAC, occurs in only a small percentage of gallstone patients. Subsequently, Rosmorduc et al., and others, [109,110,111] further studied this subgroup of patients with a rare monogenic cause of cholesterol gallstones. Their clinical findings show that the phenotypic spectrum of *ABCB4* mutations in either the homozygous or heterozygous state is associated with LPAC, including (i) clustering within families; (ii) early onset of biliary symptoms, often before the age of 40 years; (iii) gallbladder cholesterol gallstones; (iv) intrahepatic hyperechoic foci, intrahepatic sludge, microlithiasis, or macroscopic cholesterol gallstones in the intrahepatic bile ducts; and (v) a high risk of recurrence of biliary symptoms after cholecystectomy [108,109,110,111].

However, the vast majority of LPAC patients are diagnosed retrospectively, primarily due to recurrence of biliary symptoms after cholecystectomy [112]. As a consequence, fresh gallbladder or hepatic bile is not collected from these LPAC patients, before or during surgery, for chemical analysis of bile lipids. Due to some limitations of technical issues, such as collection of fresh gallbladder bile samples and early diagnosis of LPAC patients with asymptomatic gallstones, it is not possible to study cholesterol nucleation and crystallization in LPAC patients, nor to investigate the evolution of gallstone formation. Furthermore, because of the lack of information on the lipid composition of gallbladder and hepatic bile, cholesterol nucleation and crystallization, and the sequence of gallstone formation, it is difficult to decipher the pathogenesis of cholesterol gallstones caused by *ABCB4* mutations or variants in patients with LPAC.

In early in vitro studies of cholesterol nucleation and crystallization using a series of model bile systems, needle-like, arc-like, and filamentous cholesterol crystals have been identified in bile with low phospholipid/bile acid ratios, and they are found to be apparently “anhydrous” cholesterol crystals that subsequently evolve into plate-like cholesterol monohydrate crystals through a series of intermediates [24,106,107]. More importantly, similar nucleation and crystallization of putative “anhydrous” cholesterol is also found in phospholipid-deficient gallbladder bile of ABCB4 KO mice even on chow [105]. The chemical composition of fresh gallbladder and hepatic bile is compared between ABCB4 KO and WT mice, showing that gallbladder bile is supersaturated with cholesterol in the former, but not in the latter, even without a dietary challenge, i.e., not feeding the lithogenic diet. As a result, gallstones are spontaneously formed in ABCB4 KO mice on chow, as evidenced by a systematic microscopic and physical-chemical analysis of the biliary and gallstone phenotypes. These studies identify needle-like “anhydrous” cholesterol crystals and gallstone formation as an important feature of ABCB4 KO mice [105], and these exciting findings further confirm predictions from in vitro studies of model bile with low phospholipid/bile acid ratios that are similar to the lipid composition of gallbladder bile of ABCB4 KO mice [24,106,107]. More importantly, these phenotypic findings in ABCB4 KO mice even on chow are in agreement with the hepatobiliary observations of *ABCB4* mutations in patients with LPAC.

**Figure 3:**
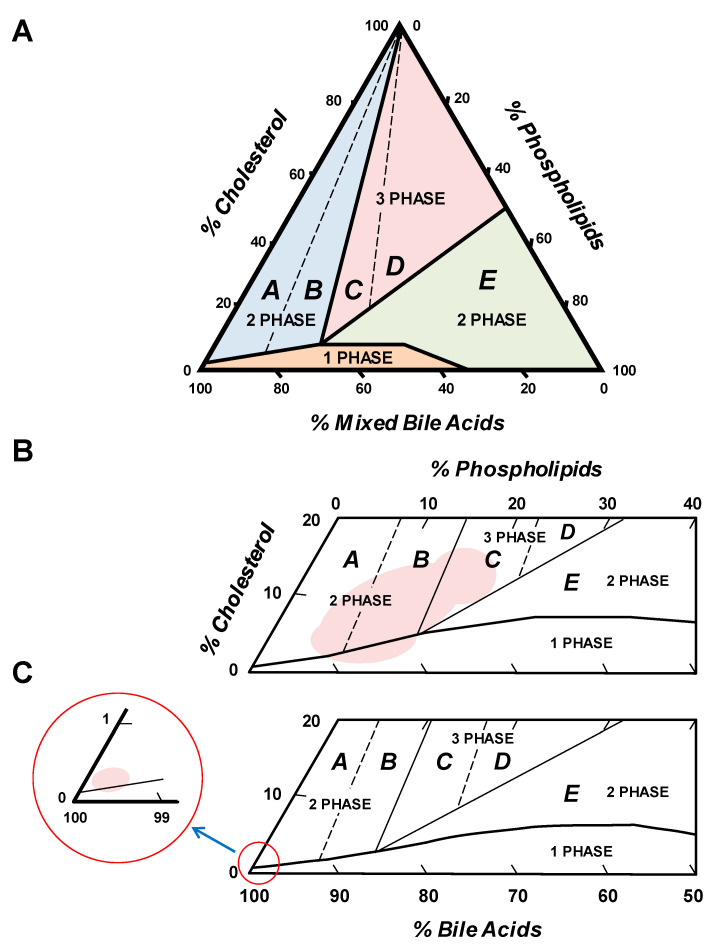
(**A**) An equilibrium phase diagram of cholesterol–phospholipid-mixed bile acid system (37 °C, 0.15 M NaCl, pH 7.0, total lipid concentration 7.5 g/dL) shows positions and configuration of crystallization regions. The components are expressed in mole percent. The one-phase micellar zone at bottom is enclosed by a solid curved line. Above it, two solid lines divide the two-phase zones from a central three-phase zone. Based upon the solid and liquid crystallization sequences present in the bile, the left two-phase and the central three-phase regions are divided by dashed lines into regions A to E. The number of phases given represents the equilibrium state. Solid plate-like cholesterol monohydrate crystals and saturated micelles are present in crystallization regions A and B. Liquid crystals, cholesterol monohydrate crystals, and saturated micelles are found in regions C and D. Liquid crystals of variable composition and saturated micelles are present in region E. (**B**) Positions of crystallization regions A–E in human gallbladder bile are shown in a condensed phase diagram. The phase boundaries are drawn according to the mean total lipid concentrations and compositions of human gallbladder bile samples. This phase diagram exhibits the same physical states at equilibrium, as described for (**A**). The pink areas show positions of relative lipid composition of gallbladder bile of ordinary gallstone patients, i.e., these without *ABCB4* mutations or variants, in equilibrium phase diagrams. (**C**) Based on the relative lipid composition of gallbladder bile in ABCB4 knockout mice on chow, this condensed phase diagram shows the predicted lipid composition of gallbladder bile of patients with low phospholipid-associated cholelithiasis (LPAC) caused by *ABCB4* mutations and variants. Of special note, due to a reduction or deficiency of phospholipids in bile, total lipid concentration could be reduced such that all crystallization pathways are shifted to lower phospholipid contents, with micellar cholesterol solubility being diminished. The predicted lipid composition of gallbladder bile of LPAC patients is mainly distributed in crystallization region A, i.e., the lower left corner of the phase diagram, as indicated by the small red circle. The area of detail illustrates the lower left corner of the phase diagram for bile with low ratios of phospholipids to bile acids, as shown by the large red circle. In this region, the gallbladder bile is characterized by low phospholipid/bile acid ratios, supersaturation with cholesterol, and precipitation of needle-like cholesterol crystals, putatively “anhydrous” cholesterol crystallization at low phospholipid concentrations in model and native bile (see text for further details).

Although *ABCB4* mutations and variations have been identified as a monogenetic risk factor for LPAC in humans and the ABCB4 KO mouse model has provided a physical-chemical explanation for the formation of gallstones, how needle-like “anhydrous” cholesterol crystals nucleate and crystallize in phospholipid-deficient bile remains elusive. Cholesterol and lecithin molecules are secreted into bile by ABCG5/G8 and ABCB4 transporters located on the canalicular membrane of hepatocytes (Figure 1). Once lecithin enters the canalicular space, it rapidly forms vesicles [77], which greatly enhance cholesterol solubility in bile. The composition of these vesicles can be inferred from the molar ratio of cholesterol to lecithin in fresh hepatic bile of ABCB4 KO and WT mice. Based on phase diagrams from the study of model bile systems, classical plate-like cholesterol monohydrate crystals often precipitate from vesicles supersaturated with cholesterol and with a cholesterol/lecithin ratio of approximately 0.50. Moreover, the molar ratio of cholesterol to lecithin is greater than 1.00, with a range being between 0.65 and 2.24 in bile of ABCB4 KO mice [105]. It is highly likely that the lipid composition of bile in LPAC patients is supersaturated with cholesterol in combination with a low concentration or a lack of phospholipids, leading to cholesterol/lecithin ratios between 0.44 and 0.93. At equilibrium, the biliary composition of pooled gallbladder bile of ABCB4 KO mice is located in the lower left crystallization region A of the phase diagram (Figure 3), which often nucleates and crystalizes needle-like, arc-like, and filamentous “anhydrous” cholesterol crystals. To understand the kinetics and characteristics and metastable intermediates in the phase transitions of bile, the phase diagrams with varying mole percentages of cholesterol, phospholipids, and bile acids have been used as templates to investigate the crystallization regions wherein different sequences of metastable intermediates take place. As shown in Figure 3, five crystallization zones from A to E have been defined, with each illustrating a different sequence of phase transitions, including a liquid crystalline and an “anhydrous” crystalline pathway [24]. Ultimately, both liquid crystalline and “anhydrous” crystalline metastable intermediates can evolve into typical cholesterol monohydrate crystals, with 79.2° and 100.8° angles, and often a notched corner. Furthermore, these crystallization pathways have been confirmed in human and mouse gallbladder bile [113,114,115].

**Figure 4:**
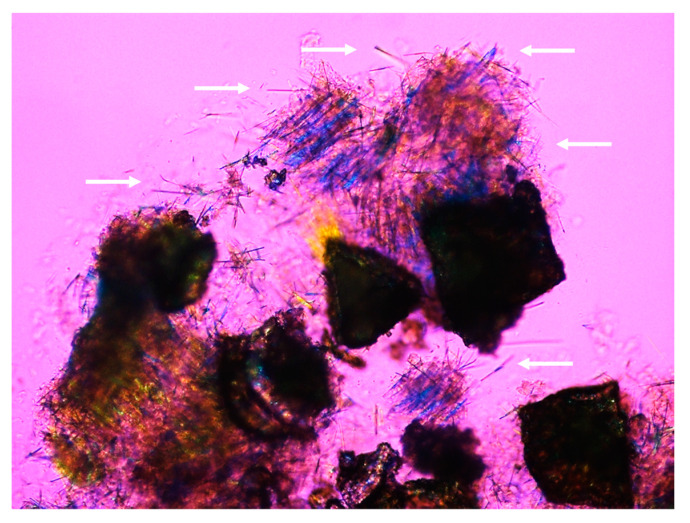
Representative photomicrographs of needle-like cholesterol crystals (arrows) and gallstones in fresh gallbladder bile of ABCB4 KO mice. The figure was obtained using phase contrast and polarizing light microscopy (magnification ×400) at room temperature (20 °C). To highlight the birefringence of the solid needle-like cholesterol crystals, the microscope was operated with crossed polars in operation without a first-order quartz compensator. Needle-like cholesterol crystals (arrows) are short, straight, filamentous cholesterol crystals that project from the edges of the brownish gallstones. Notably, liquid crystals are absent.

However, it is still an intriguing question as to why “anhydrous” cholesterol crystals precipitate from cholesterol-supersaturated bile under certain conditions, e.g., reduced phospholipid concentrations (Figure 4). Notably, it remains elusive whether these “initial” crystalline forms are truly “anhydrous” cholesterol that undergoes a polymorphic transition or represents a series of novel metastable intermediates evolving to monohydrate polymorph, as shown by a discrete diffraction pattern upon electron diffraction of single crystals in nucleating model bile of similar lipid composition to that of ABCB4 KO mice. Furthermore, the classical notched rhombohedral crystals of cholesterol monohydrate are not detected in fresh gallbladder bile of ABCB4 KO mice on chow [105]. Therefore, it is unclear whether needle-like cholesterol crystals could ultimately evolve into plate-like cholesterol monohydrate crystals through multiple crystal forms and habits, as observed in native gallbladder bile of humans and inbred mice, which include one set of intermediates such as arc-like crystals, helices, and tubular crystals of “anhydrous” cholesterol. In addition, it has become increasingly interesting to investigate the origin of needle-like and arc-like “anhydrous” cholesterol crystals. It has been hypothesized that solid “anhydrous” cholesterol crystals in ABCB4 KO mice may originate from bile acid-dissolution of residual cholesterol-rich vesicles. Another hypothesis is that in the crystallization region A (Figure 3), cholesterol molecules in vesicles supersaturated highly with cholesterol are not only phase-separated into a continuous row of molecules, but are also not yet hydrated. A third possible explanation is that there may be direct bile acid elution of structural phospholipids and cholesterol from the canalicular membrane of hepatocytes, as evidenced by increased hydrophobicity indexes of bile acids in lithogenic bile compared to cholesterol-unsaturated bile of humans and mice.

Epidemiological and clinical investigations have clearly demonstrated that intrahepatic stones, also known as hepatolithiasis, are more prevalent in Asia but are less common in Europe [116]. Although the majority of LPAC patients reported in the literature are in Europe [117], intrahepatic stones are often a common feature of the disease, suggesting a close relationship among *ABCB4* deficiency, reduction or lack of biliary phospholipids, and hepatolithiasis [108,109,110,111]. Moreover, in a group of Japanese patients with a significant reduction in expression of *ABCB4* mRNA and protein in the liver, the concentration of phospholipids in gallbladder bile is dramatically decreased such that they suffer from cholesterol gallstones and cholesterol-rich brown pigment stones in the intrahepatic bile ducts [118,119]. These studies indicate that reduced phospholipid concentrations in bile caused by either *ABCB4* mutations and variations or reduced expression can promote the formation of intrahepatic stones, regardless of whether the patient is European or Asian [112,120,121,122]. This concept is further supported by findings from another study revealing that a subgroup of patients with cholesterol microlithiasis show a significant reduction in mean percent molar concentrations of phospholipids in duodenal bile [123].

Why the prevalence of intrahepatic stones is high in LPAC patients is an intriguing question. Furthermore, the relationship between liver fibrosis and intrahepatic stones in these patients remains elusive. Does liver fibrosis contribute to the formation of needle-like “anhydrous” cholesterol crystals and intrahepatic stones, or do needle-like cholesterol crystals trigger the development of liver fibrosis and subsequently go on to exacerbate liver fibrosis in patients with LPAC? Due to limitations in collecting liver samples for pathology studies in the early stages of liver fibrosis and intrahepatic stones in LPAC patients, no clinical evidence is provided to address these issues. Using phase contrast and polarizing light microscopy of liver cryostat sections, agglomerates of solid cholesterol crystals are found in the intrahepatic bile ducts of 7-month-old ABCB4 KO mice [105]. At about the same time, however, neither stones in the liver nor solid cholesterol crystals in fresh hepatic bile samples are found in male ABCB4 KO mice. In contrast, these mice have developed fibrotic, obliterative sclerosing cholangitis at 8 weeks of age. Additionally, periductal fibrosis and other features of bile duct injury and inflammation can be observed as early as 3 weeks after birth [105]. It is likely, therefore, that hepatic bile stasis due to segmental biliary obstruction caused by intrahepatic bile duct injury, inflammation, and stricture may be associated with the formation of intrahepatic stones under conditions of an abnormal bile lipid composition, especially in those with reduced or absent biliary phospholipids and high cholesterol/lecithin ratios. Notably, needle-like cholesterol crystals have been found in some patients with acalculous gallbladder disease [124,125], and it is unclear whether there is a possible pathophysiologic link between this disease entity and low biliary phospholipid concentrations. Moreover, it is an intriguing question as to whether needle-like cholesterol crystals contribute to gallbladder and intrahepatic bile duct injury and inflammation (Figure 4). It has been found that excessive amounts of monosodium urate monohydrate and calcium pyrophosphate dihydrate often form needle-like crystals, which have a “cytotoxic” effect on triggering joint inflammation in patients with gout and pseudogout [126]. Therefore, it is interesting to speculate that a similar mechanism to this is that needle-like “anhydrous” cholesterol crystals may interact with the apical membrane of epithelial cells of the gallbladder and intrahepatic bile ducts, leading to cholecystitis and cholangitis in ABCB4 KO mice and LPAC patients.

The phenotyping study of bile and cholesterol crystallization in ABCB4 KO mice could be helpful in establishing a standard for the diagnosis of LPAC in patients because needle-like “anhydrous” cholesterol crystals, aggregated solid cholesterol crystals as bound by mucin gel, or cholesterol microlithiasis can be found by phase contrast and polarizing light microscopy in endoscopically collected hepatic or duodenal bile. More importantly, these bile samples can be found to be reduced contents of phospholipids in relation to bile acids as analyzed by chemical methods. Both would facilitate rapid and initial diagnosis of LPAC in suspected patients.

Similar to the molecular species of phospholipids, bile acid species can have an impact on cholesterol nucleation and crystallization. Notably, there is a more hydrophobic bile acid pool because of a lower concentration of tauro-β-muricholic acid in bile of female ABCB4 KO mice [105]. Increasing bile acid hydrophobicity has been shown in vitro to promote cholesterol nucleation and crystallization by diminishing the liquid crystal region E of the phase diagram, where the precipitation of solid cholesterol monohydrate crystals is a forbidden phase transition [24]. This may explain the earlier onset and higher prevalence of gallstones in female ABCB4 KO mice compared with males [105]. These findings provide a rationale for using a more hydrophilic bile acid, ursodeoxycholic acid (UDCA), to replace more hydrophobic bile acids in bile of patients with LPAC. Therefore, it is highly likely that most, but not all, patients with LPAC may benefit from long-term primary or secondary prophylactic UDCA therapy, which should be recommended for all patients at a young age [127].

However, as suggested by ABCB4 KO mice [105], a more hydrophilic bile acid pool may not be sufficient to completely prevent cholecystolithiasis and hepatolithiasis caused by phospholipid deficiency. This may even contribute to a deleterious effect on the development of liver fibrosis in the presence of fully developed strictures and mechanical obstruction of the intrahepatic bile ducts. In the future, ABCB4 KO mice are expected to serve as a useful experimental animal model to explore innovative therapeutic interventions, such as gene therapy, for human LPAC.

## 5. Genetic Analysis of *ABCB4* Mutations and Variants in Patients with Low Phospholipid-Associated Cholelithiasis (LPAC)

A small number of patients with LPAC have been reported, but the exact prevalence in the entire population is unknown. In addition, although familial genetic screening for LPAC in several cases of patients and their family members has been investigated [108,110,128,129,130] and approximately 158 mutations and variants in the *ABCB4* gene have been identified, the genetic mechanisms of LPAC have not been established. In particular, more genotyping information and more phenotype–genotype studies are needed to decipher whether LPAC may be a Mendelian disorder. However, the relationship between dysfunctional ABCB4 protein and LPAC is consistent with the increased prevalence of gallstones in PFIC3 and ICP [75,131], which is highly associated with *ABCB4* mutations and variants in these patients. Although numerous mutations and variants in *ABCB4* have been identified and reported in the literature, it is still a challenging task to perform functional studies on all identified variants to demonstrate their effects on protein expression and biological function of the gene, hepatic phospholipid output, cholesterol nucleation and crystallization, gallstone formation sequence, onset and severity of the disease, and other hepatobiliary pathology such as gallbladder dysmotility, cholestasis, and liver fibrosis and cirrhosis. Therefore, it is imperative to precisely define how *ABCB4* mutations and variants influence clinical phenotypes of LPAC patients because this may provide key insights into the mechanisms underlying the critical roles of these mutations and variants in determining the function of the encoded protein and the pathophysiology of LPAC [103].

In general, major sequence variations in genes regulating essential biochemical processes result in clinical manifestations, including the onset of symptoms and disease severity [132,133,134,135]. By contrast, minor variants have milder phenotypes or are linked to disease susceptibility [136,137,138,139,140,141]. Some findings have suggested phenotype–genotype relationships [117]; however, few studies have directly demonstrated the biological and functional impact of missense variants, the most common sequence abnormalities in patients with ABCB4 deficiency [142]. These variants in *ABCB4* have been found along the entire gene length [143]. Given the typical mutation-causing changes such as nonsense and frame shifting deletions and insertions, laboratories can use algorithms to classify variants into mutations or benign polymorphisms [144,145,146]. Moreover, nucleotide changes, such as missense variants, may impair the biological function of the encoded ABCB4 protein [147]. Table 1 lists the identified mutations and variants in the *ABCB4* gene in patients with LPAC in chronological order.

Based upon the analysis of the potential functional characterization, Delaunay and colleagues [148] have made a proposal about the classification of *ABCB4* variations from I to V, which primarily depend on whether they affect the traffic, maturation, activity, and stability of the protein. Class I, variations cause defective synthesis, predominantly nonsense and frameshift mutations. Class II, variations result in a maturation defect of the protein with retention in the endoplasmic reticulum so that the mutant ABCB4 protein is almost fully expressed as an immature form. Notably, these variations in the homozygous status seem to be particularly detrimental. Class III, variations induce defective activity of the protein, with little or no effect on maturation. The majority of variations fall into this category. Class IV, variations may impair the stability of ABCB4 protein. Or, although variants are fully processed and active, the stability of the protein is significantly reduced. Class V, while these variants influence neither the localization nor the activity or stability of ABCB4, they have no detectable biological effect. Of special note is that the proposed classification of *ABCB4* variations [148] is largely based on a quite small number of subjects studied so that there are certain limitations in the analysis of the relationship between genotype, biological effect, physiological function, and clinical phenotype of ABCB4 deficiency. Moreover, homozygotes and compound-heterozygotes for *ABCB4* variants often result in severe liver disease with higher prevalence of cirrhosis, whereas heterozygotes exhibit merely mild to moderate liver disease [149]. However, this classification does not include a genetic analysis of the heterozygous status associated with *ABCB4* mutations and variations, a particularly interesting scenario as found by the reports on adult patients with idiopathic cholestasis and liver fibrosis primarily caused by *ABCB4* heterozygosity [117]. It should be emphasized that, as shown in Table 1, most LPAC patients are heterozygous, whereas a few are homozygous. More importantly, this classification of *ABCB4* variations [148] is mainly based on the investigations of patients with PFIC3. Therefore, its validation in patients with LPAC need to be further studied.

**Table 1:** Mutations and Variants in the *ABCB4* Gene in Patients with low phospholipid-associated cholelithiasis (LPAC).

Authors (Year of Publication)	Location and Nucleotide Change	Peptide Change	Predicated Domain	Status	Ref
Rosmorduc et al. (2001)	c.523A > G	p.T175V	ICD1	N/A	[108]
	c.959C > T	p.S320F	TMD5	N/A	
	c.1327insA	p.447X	NBD1	N/A	
	c.3481C > T	p.P1161S	N/A	N/A	
Rosmorduc et al. (2003)	c.495T > A ^1^	p.F165I	ICD1	HTZ	[150]
	c.523T > C	p.T175A	TMD2/3	HTZ	
	c.902T > C	p.M301T	TMD5	HTZ	
	c.959C > T	p.S320F	TMD5	HTZ	
	c.1007-1015insT	p.355X	TMD6	HTZ	
	c.1007-1015delT	p.341X	TMD6	HTZ	
	c.1327insA	p.447X	NBD1	HTZ	
	c.1584G > C	p.E528D	NBD1	HTZ	
	c.1772T > A	p.L591Q	ICD3	HTZ	
	c.1973G > A	p.Y658X	ICD3	HTZ	
	c.2270-2273insT	p.793X	ICD4	HTZ	
	c.2363G > T	p.R788E	ICD4	HTZ	
	c.2800G > T	p.A934T	ICD5	HTZ	
	c.3481C > T	p.P1161S	NBD2	HTZ	
Lucena et al. (2003)	N/A	p.G535D	Exon 14	HZT	[128]
Kano et al. (2004)	c.537-613del	N/A	TMD3	HTZ	[121]
	c.1015del	N/A	TMD6	HTZ	
	c.2683-2924del	N/A	TMD11	HTZ	
Fein et al. (2007)	c.3683-3688del	N/A	Exon 28	HTZ	[129]
	c.1769G > A	N/A	Exon15	HTZ	
Nakken et al. (2009)	c.337A > G	p.M113L	Exon 5	HTZ	[151]
	c.523A > G	p.T175A	Exon 6	HTZ	
	c.1399-1400ins10	p.Y467F fs × 25	Exon 13	HTZ	
	c.1584G > A	p.E528D	Exon 14	HTZ	
	c.1769G > A	p.R590Q	Exon 15	HTZ	
	c.1954A > G	p.R652G	Exon 16	HTZ	
	c.3136C > T	p.R1046X	Exon 25	HTZ	
	c.3318G > C	p.Q1106H	Exon 26	HTZ	
	c.175C > T	N/A	Exon 4 ^3^	N/A	
	c.504C > T	N/A	Exon 6 ^3^	N/A	
	c.711A > T	N/A	Exon 8 ^3^	N/A	
	c.1314G > A	N/A	Exon 12 ^3^	N/A	
	c.136-76delA		Intron 3	N/A	
	c.1231-81delT		Intron 11	N/A	
	c.1231-70A > G		Intron 11	N/A	
	c.1356+26A > G		Intron 12	N/A	
	c.1359-40A > G		Intron 12	N/A	
	c.1732-37A > G		Intron 14	N/A	
	c.2211+16C > T		Intron 17	N/A	
	c.2478+40A > G		Intron 20	N/A	
	c.3486+10insA		Intron 26	N/A	
	c.3487-16T > C		Intron 26	N/A	
Poupon et al. (2010)	N/A	p.F165I	N/A	N/A	[143]
	N/A	p.T175A	N/A	N/A	
	N/A	p.D180 fs + X	N/A	N/A	
	N/A	p.M301T	N/A	N/A	
	c.959C > T	p.S320F	N/A	HTZ	
	N/A	p.V336 fs + X	N/A	N/A	
	N/A	p.Q443 fs + X	N/A	N/A	
	N/A	p.E528D	N/A	N/A	
	N/A	p.R545G	N/A	N/A	
	N/A	p.A559T	N/A	N/A	
	N/A	p.L591Q	N/A	N/A	
	N/A	p.W658X	N/A	N/A	
	N/A	p.L756 fs + X	N/A	N/A	
	N/A	p.R788E	N/A	N/A	
	N/A	p.A934T	N/A	N/A	
	c.2858C > A	p.A953D	N/A	HTZ	
	N/A	p.P1161S	N/A	N/A	
Poupon et al. (2010)	N/A	p.R47Q	Exon 4	HTZ	[110]
	N/A	p.A287V	Exon 9	HTZ	
	N/A	p.G384R	Exon 11	HTZ	
	N/A	p.R406Q	Exon 11	HTZ	
	N/A	p.V526F	Exon 14	HTZ	
	N/A	p.T775M	Exon 19	HTZ	
	N/A	p.A934T	Exon 23	HTZ	
	N/A	p.A946T	Exon 23	HTZ	
	N/A	p.Y1086X	Exon 25	HTZ	
Poupon et al. (2013)	c.139C > T	p.R47X	N/A	HTZ	[117]
	c.217C > G	p.L73V	N/A	HTZ	
	c.475C > T	p.R159X	N/A	HTZ	
	c.523A > G	p.T175A	N/A	HTZ	
	c.879delA	p.A294L fs × 14	N/A	HTZ	
	c.1015delT	p.S339Q fs × 3	N/A	HTZ	
	c.1015delT	p.S339F fs × 17	N/A	HTZ	
	c.1326dup	p.R444E fs × 5	N/A	HTZ	
	c.1420delG	p.V474W fs × 2	N/A	HTZ	
	c.1553delT	p.L518V fs × 16	N/A	HTZ	
	c.1584G > C	p.E528D	N/A	HTZ	
	c.1769G > A	p.R590Q	N/A	HTZ	
	c.2273dup	p.F760I fs × 34	N/A	HTZ	
	c.2406G > A	p.W802X	N/A	HTZ	
	c.2662G > T	p.E888X	N/A	HTZ	
	c.3258C > A	p.Y1086X	N/A	HTZ	
	c.3359C > T	p.R1187X	N/A	HTZ	
	c.3683-3688delTGATTG	p.V1228-I1229del	N/A	HTZ	
	c.139C > G	p.R47G	Nter	HTZ	
	c.140G > A	p.R47Q	Nter	HTZ	
	c.212T > A	p.L71H	TMD1	HTZ	
	c.217C > G	p.L73V	TMD1	HTZ	
	c.233T > G	p.F78C	TMD1	HTZ	
	c.296C > T	p.S99F	ECD1	HTZ	
	c.370G > A	p.G124S	TMD2	HTZ	
	c.461T > C	p.F154S	ICD1	HTZ	
	c.493T > A	p.F165I	ICD1	HTZ	
	c.523A > G	p.T175A	ICD1	HTZ	
	c.833G > A	p.R278K	ICD2	HTZ	
	c.857C > T	p.A286V	ICD2	HTZ	
	c.902T > C	p.M301T	TMD5	HTZ	
	c.959C > T	p.S320F	TMD5	HTZ	
	c.959C > T	p.S320F	TMD5	HMZ	
	c.1217G > A	p.R406Q	ICD3	HTZ	
	c.1529A > G	p.N510S	ICD3/NBD1	HTZ	
	c.1531G > A	p.A511T	ICD3/NBD1	HTZ	
	c.1537G > A	p.E513K	ICD3/NBD1	HTZ	
	c.1584G > C	p.E528D	ICD3/NBD1	HTZ	
	c.1621A > T	p.I541F	ICD3/NBD1	HTZ	
	c.1634G > A	p.R545H	ICD3/NBD1	HTZ	
	c.1646G > A	p.R549H	ICD3/NBD1	HTZ	
	c.1675C > A	p.A559Y	ICD3/NBD1	HTZ	
	c.1765C > T	p.H589T	ICD3	HTZ	
	c.1769G > A	p.R590Q	ICD3	HTZ	
	c.1769G > A	p.R590Q	ICD3	HMZ	
	c.1772T > A	p.L591Q	ICD3	HTZ	
	c.1778C > A	p.T593M	ICD3	HTZ	
	c.1939G > A	p.E647K	ICD3	HTZ	
	c.2177C > T	p.P726L	TMD7	HTZ	
	c.2186C > T	p.S729L	TMD7	HTZ	
	c.2363G > A	p.R788Q	ICD4	HTZ	
	c.2800G > A	p.A934T	ICD5	HTZ	
	c.2800G > A	p.A934T	ICD5	HMZ	
	c.2923C > G	p.L975V	ECD6	HTZ	
	c.3250C > T	p.W1084W	NBD2/Cter	HTZ	
	c.3481C > T	p.P1161S	Cter	HTZ	
	c.3481C > T	p.P1161S	NBD2/Cter	HTZ	
Kim et al. (2013)	c.495T > A ^2^	p.F165I	ICD1	N/A	[152]
	c.902T > C	p.M301T	TM5	N/A	
	c.959C > T	p.S320F	TM5	N/A	
Condat et al. (2013)	c.127G > A	p.V43I	Exon 4	HTZ	[153]
	c.1006-162_1119+706del	p.V336_N373del	Exon 10	HTZ	
	c.1529A > G	p.N510S	Exon 13	HTZ	
	c.1331T > C	p.V444A	Exon 13	HMZ	
	c.1769G > A	p.R590Q	Exon 15	HTZ	
Jirsa et al. (2014)	c.147C > T	p.S49S	N/A	N/A	[154]
	c.175C > T	p.L59L	N/A	N/A	
	c.459T > C	p.F153F	N/A	N/A	
	c.504C > T	p.N168N	N/A	N/A	
	c.523A > G	p.T175A	N/A	HTZ	
	c.711A > T	p.I237I	N/A	N/A	
	c.1371delG	p.Q458R fs × 7	N/A	HTZ	
	c.1954A > G	p.R652G	N/A	HTZ	
	c.2222C > T	p.P741L	TMD7/8	HTZ	
	c.2318G > T	p.G773V	TMD8	HTZ	
Gudbjartsson et al. (2015)	N/A	p.L445G fs × 22	N/A	N/A	[155]
	N/A	p.N510S	N/A	N/A	
	N/A	p.G622E	N/A	N/A	
	c.711A > T	N/A	N/A	N/A	
Reichert and Lammert (2018)	c.139T > C	N/A	N/A	HTZ	[156]
	c.959T > C	N/A	N/A	HTZ	
Schatz et al. (2018)	c.523A > G	p.T175A	N/A	HTZ	[157]
	c.1731G > A	Splicing variant	N/A	HTZ	
	c.1744C > T	p.R582W	N/A	HTZ	
	c.1769G > A	p.R590Q	N/A	HTZ	
	c.2380G > C	p.A794P	N/A	HTZ	
	c.2507C > A	p.A836E	N/A	HTZ	

Abbreviations: Cter, C-terminal; del, deletion; dup, duplication; ECD, extracellular domain; fs, frameshift; HMZ, homozygous; HTZ, heterozygous; ICD, intracellular domain; ins, insertion; N/A, not available; NBD, nucleotide binding domain; Nter, N-terminal; Ref, references; TMD, transmembrane domain; X, stop; Xn, stop after n codons. ^1^ The A of ATG of the initiator Met codon is denoted as “nucleotide + 1”. ^2^ Position of each mutant is based upon the translational start site. ^3^ All these four synonymous exon variations found are described in the National Center for Biotechnology Information (NCBI) dbSNP (https://www.ncbi.nlm.nih.gov/SNP/snp_ref.cgi?locusld=5244 accessed on 22 May 2022).

The American College of Medical Genetics and Genomics and the Association for Molecular Pathology have jointly proposed the standards and guidelines for the classification and interpretation of sequence variants from benign to pathogenic in a Mendelian disorder [158]. A challenge for clinical laboratory tests characterizing the pathogenicity of *ABCB4* mutations and variants is that individuals with *ABCB4* mutations or variants often suffer from a range of liver diseases whose clinical manifestations are closely related to the dysfunctional ABCB4 protein. This highlights the possibility that individuals with the same *ABCB4* genotype could display different clinical phenotypes. Notably, most cases harbor only one mutation or variant, while a few contain two mutations and/or variants [117]. It remains elusive whether there is a difference in the onset of symptoms and the course and severity of the disease between these two groups of LPAC patients. In addition, interactions between *ABCB4* variants and environmental factors may promote clinical manifestations with variable phenotypes. This may explain why ABCB4-associated liver diseases display diverse clinical features, ranging from LPAC to PFIC3 and from ICP to liver fibrosis and cirrhosis [159,160,161]. The distribution of ABCB4 mutations and variants in LPAC, PFIC3 and ICP differed but did not overlap [143]. Only a few mutations and variants are shared between LPAC, PFIC3, and ICP [128]. In addition, it has been noted that some cases with the same *ABCB4* genotype suffer from different types of liver disease, which could be LPAC, PFIC3, or LPC, whose phenotypic characteristics depend on the age of onset [157]. Therefore, conclusions about the roles of *ABCB4* mutations and variants in the pathogenesis and pathophysiology of these liver diseases in a clinical setting should be interpreted carefully and thoughtfully.

As it is not possible to perform functional studies on all identified mutations and variants to demonstrate the effect on the formation of ABCB4 protein, in silico predictive tools are likely to be a key component in determining the pathogenicity of *ABCB4* mutations and variants [162,163,164]. Basically, these computational programs rely largely on a variety of algorithms and are mainly useful for predicting the effect of missense changes on the underlying biological function of the ABCB4 protein and to a lesser extent on splicing [165]. However, they have limitations in accuracy, especially when interpreting conclusions about the effects of novel *ABCB4* variants.

Notably, inactivation of nonsense-mediated mRNA decay may result in the expression of the ABCB4 protein with possibly residual function, which may explicate the alleviated disease course in some, but not all, patients with homozygous frameshift variants [166]. Similar to many monogenic diseases, genotype–phenotype studies are not helpful in understanding disease mechanisms. Variability in the clinical phenotype is a common phenomenon in human genetic diseases. In addition, the onset of symptomatic gallstones is different from the development of gallstone disease in patients with LPAC. This could be explained by the fact that approximately two thirds of ordinary gallstone patients, i.e., those without *ABCB4* mutations or variants, have been found to be asymptomatic in the clinical setting [167]. It remains unclear why there is a striking difference in disease severity of LPAC even among members of the same family. It has been observed that in certain cases, LPAC patients harboring a *ABCB4*-causing mutation or variant can be completely asymptomatic throughout their whole life. It is also common in some patients with gallstones diagnosed only at autopsy [167]. In principle, this is a genetic phenomenon called incomplete penetrance [132].

In summary, there is a complex interaction between genetic and environmental factors such as diets, gender, hormones, aging, excise, insulin resistance, obesity, diabetes, and drugs, especially in rare genetic diseases with broad phenotypic expression, such as LPAC and inherited cholestatic liver diseases such as PFIC3, with both having numerous mutations and variants. Therefore, genetic testing, including in silico computational prediction of pathogenicity of *ABCB4* mutations and variants, is only one step in the diagnostic and therapeutic decision-making process. In addition, it is strongly recommended that LPAC patients and their siblings, even asymptomatic, perhaps harboring novel *ABCB4* mutations and variants, need a complete diagnostic workup including laboratory, imaging, and histological examinations of the hepatobiliary system for a careful and thorough evaluation of the genotype and phenotype of ABCB4 deficiency.

## 6. Future Research Directions and Clinical Applications

The identification of susceptible subjects and potential patients with LPAC is a challenging task. To date, no cases of LPAC with preoperative diagnosis have been reported. In contrast, almost all LPAC cases were diagnosed retrospectively, suggesting that preoperative diagnosis is not easy. If LPAC is suspected in young (age < 40 years) patients with gallstones, genetic analysis of *ABCB4* mutations and variants should be performed as early as possible. It is helpful for genetic diagnosis of LPAC by sequencing all exons of the gene, which may also uncover causally relevant *ABCB4* mutations and variants. In addition, clinical diagnosis of LPAC in suspected cases may be confirmed by microscopic examination of endoscopically sampled gallbladder or duodenal bile through phase contrast and polarizing light microscopy. It is helpful for making a clinical diagnosis of LPAC if the collected bile contains needle-like “anhydrous” cholesterol crystals, aggregated solid cholesterol crystals bound by mucin gels, biliary sludge, and/or microlithiasis. More importantly, lipid analysis of these bile samples can reveal a reduction or deficiency in phospholipids in relation to bile acids.

For symptomatic gallstones, cholecystectomy plus common bile duct exploration should be performed, and in the case of extensive hepatolithiasis, hepatectomy must be considered. Endoscopic retrograde cholangiography may be helpful in the preoperative diagnosis and treatment of common bile duct stones, i.e., choledocholithiasis, and the postoperative diagnosis and management of residual gallstones in the common bile duct and/or hepatic bile ducts.

If LPAC patients with symptomatic gallstones undergo cholecystectomy, postoperative recurrence of gallstones must be prevented because the lithogenic state of bile remains due to *ABCB4* mutations and variants. In addition, the progression and prognosis of liver fibrosis and cirrhosis in LPAC patients should be monitored. Therefore, the use of the hydrophilic bile acid, UDCA, and the intestinal cholesterol absorption inhibitor, Ezetimibe, is strongly recommended for postoperative patients with LPAC [168,169,170]. It has been found that ezetimibe can prevent the formation of cholesterol gallstones by inhibiting intestinal cholesterol absorption in gallstone-susceptible mice even fed a lithogenic diet [171]. As a result, hepatic secretion of biliary cholesterol is dramatically reduced, and gallbladder motility function is preserved mainly due to bile desaturation. Combination therapy of UDCA and ezetimibe can decrease the supersaturation of cholesterol in gallbladder bile and alter the bile acid species in favor of the formation of a more hydrophilic bile acid pool, thus reducing the lithogenic state of bile. Moreover, UDCA and ezetimibe can promote postoperative dissolution of residual cholesterol gallstones in the bile ducts by two distinct physical–chemical mechanisms through the formation of a liquid crystalline mesophase and an unsaturated micelle, respectively [171].

More recently, a new method with genetically modified mRNA variants encoding human *ABCB4* (*hABCB4* mRNA) encapsulated in lipid nanoparticles for gene therapy of dysfunctional ABCB4 has been developed in a BALB/c.Abcb4 KO mouse model of PFIC3 [172]. The results from the study with this mouse model have provided clear evidence that treatment with liver-targeted *hABCB4* mRNA leads to the expression of functional hABCB4 protein in the liver, thereby restoring hepatic phospholipid secretion into bile in the mouse model of PFIC3. More importantly, synthetic *hABCB4* mRNA therapy also promotes favorable hepatocyte-driven liver regeneration to recover not only hepatic phospholipid secretion, but also normal liver function in PFIC3 mice. Therefore, mRNA therapy may be a potential option to restore biliary phospholipid content and a promising strategy for preventing or treating cholesterol gallstones by recovering normal liver function and hepatic phospholipid secretion in LPAC patients.

## Data Availability

Not applicable.
